# Induction of apoptosis and activation of the caspase cascade by anti-EGF receptor monoclonal antibodies in DiFi human colon cancer cells do not involve the c- *jun* N-terminal kinase activity

**DOI:** 10.1054/bjoc.2000.1201

**Published:** 2000-05-18

**Authors:** B Liu, M Fang, M Schmidt, Y Lu, J Mendelsohn, Z Fan

**Affiliations:** Department of Experimental Therapeutics, The University of Texas M. D. Anderson Cancer Center, Houston, Texas, 77030, USA

**Keywords:** epidermal growth factor receptor, monoclonal antibody, apoptosis, caspase, c- *jun* N-terminal kinase-1

## Abstract

We previously reported that exposure of DiFi human colon cancer cells to the anti-epidermal growth factor (EGF) receptor monoclonal antibody (mAb) 225 resulted in apoptosis, but the mechanisms remain to be elucidated. In the present study, we investigated the effects of a panel of four anti-EGF receptor mAbs, each of which binds to different epitopes of the EGF receptor in DiFi cells, on the induction of apoptosis. We found that each of these mAbs induced apoptosis in DiFi cells. Exposure of DiFi cells to mAb 225 activated the initiation caspase-8, which was detectable between 8 and 16 h after exposure of the cells to the antibody. There was also an activation of the initiation caspase-9, which lagged a few hours behind the activation of caspase-8. Exposure of DiFi cells to mAb 225 also activated the execution caspase-3, which was accompanied temporally by evidence of cleavage of a well-characterized caspase-3 substrate, poly(ADP)-ribosepolymerase (PARP). Pre-exposure of the cells to the caspase-3-specific inhibitor DEVD-CHO partially reduced the mAb 225-induced PARP cleavage and apoptosis, whereas pre-exposure of the cells to the caspase pan-inhibitor z-VAD-fmk completely inhibited mAb 225-induced apoptosis. Caspases-3, -8 and -9 were not activated in the cell lines in which mAb 225 only induced G1 phase arrest of the cell cycle. In contrast to the apoptosis of DiFi cells induced by ultraviolet irradiation, which strongly activated the c- *jun* N-terminal kinase-1 (JNK1) and the caspase cascade, mAb 225-induced apoptosis and activation of the caspase cascade in DiFi cells were not associated with activation of JNK1. © 2000 Cancer Research Campaign


*British Journal of Cancer* (2000) **82**(12), 1991–1999
© 2000 Cancer Research Campaign
doi: 10.1054/ bjoc.2000.1201, available online at http://www.idealibrary.com on IDEAL®

### Induction of apoptosis and activation of the caspase cascade by anti-EGF receptor monoclonal antibodies in DiFi human colon cancer cells do not involve the c-*jun* N-terminal kinase activity

**B Liu, M Fang, M Schmidt, Y Lu, J Mendelsohn and Z Fan**

Department of Experimental Therapeutics, The University of Texas M. D. Anderson Cancer Center, Houston, Texas 77030, USA

**Summary** We previously reported that exposure of DiFi human colon cancer cells to the anti-epidermal growth factor (EGF) receptor monoclonal antibody (mAb) 225 resulted in apoptosis, but the mechanisms remain to be elucidated. In the present study, we investigated the effects of a panel of four anti-EGF receptor mAbs, each of which binds to different epitopes of the EGF receptor in DiFi cells, on the induction of apoptosis. We found that each of these mAbs induced apoptosis in DiFi cells. Exposure of DiFi cells to mAb 225 activated the initiation caspase-8, which was detectable between 8 and 16 h after exposure of the cells to the antibody. There was also an activation of the initiation caspase-9, which lagged a few hours behind the activation of caspase-8. Exposure of DiFi cells to mAb 225 also activated the execution caspase-3, which was accompanied temporally by evidence of cleavage of a well-characterized caspase-3 substrate, poly(ADP)-ribosepolymerase (PARP). Pre-exposure of the cells to the caspase-3-specific inhibitor DEVD-CHO partially reduced the mAb 225-induced PARP cleavage and apoptosis, whereas pre-exposure of the cells to the caspase pan-inhibitor z-VAD-fmk completely inhibited mAb 225-induced apoptosis. Caspases-3, -8 and -9 were not activated in the cell lines in which mAb 225 only induced G1 phase arrest of the cell cycle. In contrast to the apoptosis of DiFi cells induced by ultraviolet irradiation, which strongly activated the c-*jun* N-terminal kinase-1 (JNK1) and the caspase cascade, mAb 225-induced apoptosis and activation of the caspase cascade in DiFi cells were not associated with activation of JNK1. © 2000 Cancer Research Campaign

**Keywords:** epidermal growth factor receptor; monoclonal antibody; apoptosis; caspase; c-*jun* N-terminal kinase-1

The receptor for epidermal growth factor (EGF) is a transmembrane glycoprotein with intrinsic tyrosine kinase activity (Ullrich and Schlessinger, 1990). Many human cancers of epithelial origin express high numbers of EGF receptors and are stimulated by activation of the receptor via a transforming growth factor-α (TGF-α)/EGF receptor autocrine loop (Ozanne et al, 1986; Cowley et al, 1986; Ishitoya et al, 1989; Hendler and Ozanne, 1984). The increased receptor levels are associated with poor clinical prognosis in patients with cancers of the bladder (Neal et al, 1985), breast (Sainsbury et al, 1985), and lung (Veale et al, 1987) and others. Blockade of EGF receptor as an approach for anticancer therapy has been extensively studied over the past decade (Fan and Mendelsohn, 1998).

Monoclonal antibody (mAb) 225 binds to EGF receptor with an affinity similar to that of the natural ligands EGF and TGF-α, competes with these natural ligands for receptor binding, and inhibits ligand-induced activation of the receptor tyrosine kinase (Sato et al, 1983; Gill et al, 1984). This antibody, along with other EGF receptor-blocking mAbs, inhibits the proliferation of a variety of cultured and xenografted tumour cell lines that are stimulated by the TGF-α/EGF receptor autocrine loop, including cell lines derived from cancers of the vulva (Fan et al, 1993*a*; 1994), breast (Ennis et al, 1989), prostate (Peng et al, 1996; Hofer et al, 1991), ovary (Ye et al, 1999), bladder (Perrotte et al, 1999), kidney (Prewett et al, 1998), lung (Lee et al, 1992), colon (Karnes et al, 1992; Wu et al, 1996), brain (Wersall et al, 1997), and head and neck (Modjtahedi et al, 1993). It has been postulated that these antibodies inhibit tumour cell proliferation by an antibody-mediated interruption of the autocrine activation of EGF receptors in cancer cells (Van de Vijver et al, 1991; Fan et al, 1993b; Baselga et al, 1996). The antibody-mediated growth inhibition in most malignant cell lines has been attributed primarily to a reduced proliferation rate with an increased cell population in G1 phase of the cell cycle (Fan et al, 1997; Wu et al, 1996; Peng et al, 1996). In nonmalignant epithelial cells there was a prominent cytostatic effect with complete G1 arrest (Stampfer et al, 1993; Chou et al, 1999).

DiFi colon cancer cells are unique in that exposure of these cells to mAb 225 resulted in not only G1 phase arrest of cell cycle, with a concomitant increase in the level of $p27^{Kip1}$ and a decrease in CDK activity, but also subsequent cell death through apoptosis (Wu et al, 1995; 1996). When nude mice bearing well-established subcutaneous DiFi cell xenografts were treated with mAb 225, the tumour was completely eradicated, a result not normally seen with other cancer cells stimulated by the TGF-α/EGF receptor autocrine pathway (Masui et al, 1991). The EGF receptor gene in these cells was not found to be rearranged or genetically altered, but the gene was found to be amplified in the range of 60–80 copies per cell, which is approximately twice the copy number

*Received 5 October 1999*
*Revised 18 February 2000*
*Accepted 19 February 2000*

*Correspondence to:* Z Fan seen in A431 epidermoid carcinoma cells (Untawale et al, 1993). DiFi cell cultures in log-phase growth secrete measurable amounts of TGF-α (347 pg per $10^6$ cells per 24 h) into their culture medium and have approximately $4.8 \times 10^6$ receptors per cell (Gross et al, 1991; Untawale et al, 1993). Although the apoptosis induced by the anti-EGF receptor mAb 225 is well documented, it is not known whether mAb 225 induces apoptosis by specifically blocking EGF receptor function or whether the apoptosis results from an incidental cross-reaction of mAb 225 with an unidentified cell surface protein, binding of which by mAb 225 can trigger an apoptotic pathway in DiFi cells. Additionally, the intracellular biochemical pathways by which mAb 225 induces apoptosis are still poorly understood.

Caspases are a family of cysteinyl aspartate-specific proteinases that mediate highly specific proteolytic cleavage events in dying cells. Although caspase-independent apoptosis may exist (Xiang et al, 1996; Susin et al, 1999), several lines of evidence indicate that caspases are critical for apoptosis in most cases. Caspase activation correlates with the onset of apoptosis, and caspase inhibition attenuates apoptosis (Bump et al, 1995; Cohen, 1997; Tewari et al, 1995). *C. elegans* mutants lacking the worm caspase CED-3 have a complete absence of developmental programmed cell death (Ellis and Horvitz, 1986). Animals deficient in caspase-3, -8 or -9 die perinatally because of profound defects in developmental programmed cell death (Green, 1998). Targeted deletion of caspase genes has shown a definitive role for caspase in apoptosis and inflammation (Green, 1998). To understand the mechanism by which mAb 225 induces apoptosis, it is essential to explore whether and how the caspase cascade is involved in the antibody-induced apoptosis.

In the current study, we examined a panel of four different mAbs (14E1, 108, 528 and 225) directed against individually distinct epitopes on the EGF receptors for their effects on inducing apoptosis. We found that each of the mAbs induced apoptosis in DiFi cells. We further demonstrated the involvement of caspases-3, -8 and -9 in mAb 225-induced apoptosis. Because mAb 225 binds to and blocks EGF receptor function and because activation of the c-*jun* N-terminal kinase-1 (JNK1) pathway with a concomitant repression of the MAP kinase cascade may result in apoptosis under certain circumstances, we therefore also investigated whether the JNK1 signaling pathway was involved in mAb 225-mediated activation of the caspase cascade and apoptosis in DiFi cells. We found that, while ultraviolet (UV) irradiation of DiFi cells strongly activated JNK1 and subsequently activated the caspase cascade and induced apoptosis, exposure of DiFi cells to mAb 225 did not activate JNK1.

### MATERIALS AND METHODS

### Materials

Anti-EGF receptor mAbs 225, 528, 108, and anti-HER2 mAb 4D5 (which we used as a control) were previously described (Fan et al, 1993*c*; Ye et al, 1999). Anti-EGF receptor mAb 14E1 was kindly provided by the Ernst Schering Research Foundation (Berlin, Germany). Anti-poly (ADP-ribose) polymerase (PARP) mAb (clone C-2–10) was purchased from CHUL Research Center, Laval University (Quebec, Canada). Anti-caspase-3 mAb (clone 19) was obtained from Transduction Laboratories (Lexington, KY, USA). Anti-JNK1 mAb (clone G151–333) was purchased from PharMingen (San Diego, CA, USA). DEVD-CHO and z-VAD-fmk were obtained from CalBiochem Corp. (San Diego, CA, USA). Caspase-3 substrate Ac-DEVD-pNA, caspase-8 substrate Ac-IETD-pNA, and caspase-9 substrate Ac-LEHD-pNA were from Alexis Corp. (San Diego, CA, USA). Protein A-Sepharose beads used for immunoprecipitation were purchased from Repligen Corp. (Cambridge, MA, USA). All other reagents were purchased from Sigma Chemical (St. Louis, MO, USA) unless otherwise specified.

### Cells and tissue culture

DiFi human colorectal adenocarcinoma cells were described previously (Wu et al, 1995; 1996). The cells were maintained in a mixture of Dulbecco's modified Eagle's medium and Ham's F-12 medium (1:1, v/v) supplemented with 10% fetal bovine serum (FBS) in a 37°C humidified atmosphere containing 95% air and 5% $CO_2$ and were split twice a week.

### Clonogenic assay

DiFi cells were seeded into 60 mm dishes at a density of 1000 cells per dish in 5 ml of medium containing 10% FBS and cultured for 15 days in a 37°C humidified atmosphere containing 95% air and 5% $CO_2$. The cell clones were stained for 5 min with a solution containing 0.5% crystal violet and 25% methanol, followed by three rinses with tap water to remove excess dye.

### Analysis of DNA fragmentation

Cells (approximately $1 \times 10^6$) were lysed with 200 μl of lysis buffer (50 mM Tris, pH 8.0, 10 mM EDTA, 0.5% N-lauroyl sarcosine, and 0.5 mg $ml^{-1}$ proteinase K). The lysates were then incubated in a 50°C water bath for 1 h prior to the addition of heat-boiled RNase A to the final concentration of 0.5 mg $ml^{-1}$; the lysates were then incubated for an additional hour in the 50°C water bath. After the digestion, the samples were diluted with an equal volume of TE buffer (10 mM Tris, pH 8.0, 1 mM EDTA). DNA was extracted three times with phenol-chloroform-isoamyl alcohol (25:24:1, v/v/v) and precipitated with 0.2 × volume of 3 M ammonia acetate and 2 × volumes of ethyl alcohol. The DNA precipitates were dissolved in TE buffer, and 5 μg of DNA from each sample was analyzed by 1.5% agarose gel electrophoresis.

### Quantification of apoptosis by ELISA

We used an apoptosis ELISA kit (Roche Diagnostics Corp., IN, USA) to quantitatively measure cytoplasmic histone-associated DNA fragments (mononucleosomes and oligonucleosomes) after induced cell death. This photomeric enzyme immunoassay was performed exactly according to the manufacturer's instructions.

### Scanning and transmission electron microscopy

DiFi cells were rinsed in serum-free medium to remove unbound protein and were then treated with a fixative containing 3% glutaraldehyde and 2% paraformaldehyde in 0.1 M cacodylate buffer, pH 7.3 (Ted Pella, Inc., Redding, CA, USA) for 1 h at ambient temperature. After fixation, the cells were washed three times with 0.1 M Millipore-filtered cacodylate-buffered tannic

*© 2000 Cancer Research Campaign*

acid, pH 7.3, and then post-fixed with 1% cacodylate-buffered osmium tetroxide (Electron Microscopy Sciences, Ft. Washington, PA, USA) for 1 h. The samples were prepared by standard procedures and examined in a Hitachi S-520 scanning electron microscope at an acceleration voltage of 5 kV and in a JEOL 1200-EX transmission electron microscope at an acceleration voltage of 80 kV.

### Caspase enzymatic activity assay

Caspase enzymatic activities were measured by colourimetric assays with a kit purchased from Clontech Laboratories, Inc. (Palo Alto, CA, USA). The assay is based on spectrophotometric detection of the chromophore *p*-nitroanilide (*p*NA), which is cleaved from caspase-specific substrates by activated caspases (DEVD-pNA by activated caspase-3, IETD-*p*NA by activated caspase-8, and LEHD-*p*NA by activated caspase-9). The assay was performed exactly according to the manufacturer's instructions.

### Terminal deoxynucleotidyl transferase-mediated dUTP-X nick end labelling (TUNEL) assay

DiFi cells were harvested and fixed in 2% formaldehyde on ice for 15 min; the fixing solution was then removed and the cell pellets were washed once with PBS. Subsequently, the cells were post-fixed in ice-cold 70% ethanol for 30 min, followed by washing the cells once with PBS and incubating the cells in 50 µl of terminal deoxynucleotidyl transferase (TdT) reaction solution containing 5 units of TdT, 0.5 nmol of biotin-dUTP, and 2.5 mM $CoCl_2$ in TdT buffer (purchased from Roche Diagnostics Corp.) at 37°C for 1 h. After the reaction, the cells were stained with a buffer containing 2.5 µg $ml^{-1}$ FITC-avidin, 0.1% Triton X-100, 5% dried low-fat milk in 4 × SSC (0.6 M sodium chloride, 60 mM sodium citrate, pH 7) in the dark at room temperature for 1 h. Before flow cytometric analysis with a FACScan flow cytometer (Becton Dickinson, San Jose, CA, USA), the cells were counterstained with a solution containing 5 µg $ml^{-1}$ propidium iodide and 10 µg $ml^{-1}$ RNase A in PBS. The FITC signal was analysed with Epics Elite software (Coulter Corp., Miami, FL, USA).

### Western blot analysis

Equal amounts of protein were used for western blot analysis with the indicated antibodies. Briefly, cells were lysed in a lysis buffer containing 50 mM Tris, pH 7.4, 50 mM NaCl, 0.5% NP-40, 50 mM NaF, 1 mM $Na_3VO_4$, 1 mM phenylmethylsulfonyl fluoride, 25 µg $ml^{-1}$ leupeptin, and 25 µg $ml^{-1}$ aprotinin. The lysates were centrifuged at the full speed of a microcentrifuge for 15 min and the supernatants were collected for protein concentration determination, SDS-PAGE, and western blot analysis with specific antibodies as described in the figure legends.

### Purification of GST-c-*jun* fusion protein

DH5α bacteria transformed with plasmid pGEX-c-*jun* (kindly provided by Dr B Su at The University of Texas MD Anderson Cancer Center, Houston, TX, USA) were grown in LB medium. After induction with 0.1 mM isopropyl-β-D-thiogalactopyranoside (IPTG) for 2 h, the bacteria were harvested and resuspended in EBC lysis buffer (20 mM Tris, pH 7.4, 50 mM NaCl, 0.5% NP-40, 1% Triton X-100) containing 0.1 µg $ml^{-1}$ leupeptin, 0.04 U $ml^{-1}$ aprotinin, and 1 mM phenylmethylsulphonyl fluoride. The bacteria were sonicated three times at 4°C for 10 s each time and then centrifuged at the full speed of a microcentrifuge at 4°C for 5 min. The supernatants were transferred to a new tube with the addition of 100 µl of 50% glutathione sepharose 4B slurry (Pharmacia Biochech Inc., Piscataway, NJ, USA). The mixture was incubated on a rotator at 4°C for 2 h, and the Sepharose 4B beads were then collected by half-full speed microcentrifugation for 15–30 s and then washed with EBC lysis buffer three times. The GST-c-*jun* fusion protein was eluted by an elution buffer (10 mM glutathione, 50 mM Tris, pH 8.0) and stored at –80°C in aliquots.

### JNK1 kinase assay

Cells were lysed with a buffer containing 50 mM Tris, pH 7.4, 50 mM NaCl, 0.5% NP-40, 50 mM NaF, 1 mM $Na_3VO_4$, 1 mM phenylmethylsulfonyl fluoride, 25 µg $ml^{-1}$ leupeptin, and 25 µg $ml^{-1}$ aprotinin. JNK1 was immunoprecipitated from 50 µg of lysate with 1 µg of specific anti-JNK1 mAb (clone G151–333) at 4°C for 3 h and with a 20 µl bed volume of protein A-Sepharose for another 3 h. The immunoprecipitates were washed three times with the lysis buffer and twice with a kinase buffer (20 mM Tris, 7.5 mM $MgCl_2$, 1 mM dithiothreitol). The kinase reaction was performed by incubating the immunoprecipitates with 40 µl of kinase buffer containing 2 µg of GST-c-*jun*, 25 µM lithium ATP, and 5 µCi of [$\gamma$-$^{32}$P]ATP (New England Nuclear, Boston, MA, USA) at 30°C for 30 min. The reaction was terminated by boiling the samples with 40 µl of 2 × SDS sample buffer. The products of the reaction were resolved using 10% SDS-PAGE and subjected to autoradiography.

### RESULTS

### Apoptosis induced by mAbs directed against the EGF receptors and its characteristics

In 1995, we first reported that exposure of DiFi cells to mAb 225 resulted in cell death through apoptosis (Wu et al, 1995). To further explore the generality of the apoptosis induced by anti-EGF receptor antibodies, we examined the effects of a panel of four different anti-EGF receptor mAbs (14E1, 108, 528 and 225) on the induction of apoptosis in DiFi cells. These antibodies bind to individually distinct epitopes on the extracellular domain of the EGF receptor and block the binding of natural ligands to the receptor (Fan et al, 1993*c*; Schmidt et al, 1997). As a control, we used the mAb 4D5 (Herceptin), which is directed against the extracellular domain of HER-2, a structurally similar receptor of the EGF receptor superfamily. Cell death was observed 24 h after the cells were exposed to these antibodies in medium containing 0.5% FBS. Treated and untreated cells were lysed, and chromosomal DNA was prepared for agarose gel electrophoresis analysis to demonstrate the characteristic DNA fragmentation (ladder) (Wu et al, 1995). We found that exposure of the DiFi cells to any of the four anti-EGF receptor mAbs produced a characteristic DNA ladder, which was not observed in the untreated cells or in cells treated with the anti-HER-2 mAb 4D5 (Figure 1A).

We further characterized the mAb dose- and time-dependency of mAb 225-induced apoptosis. The mAb 225-induced apoptosis

*© 2000 Cancer Research Campaign* 

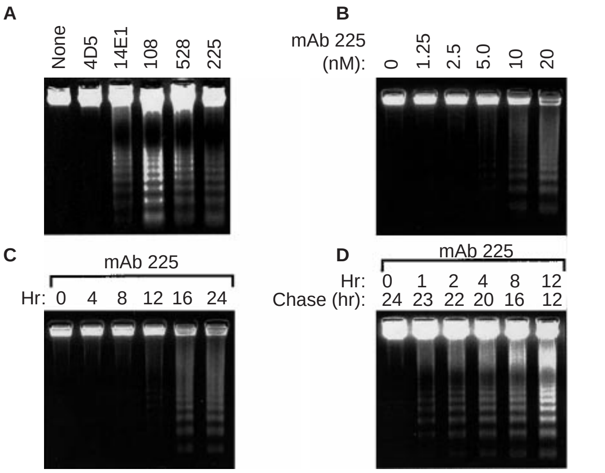

**Figure 1** Induction of DNA fragmentation by anti-EGF receptor mAbs in DiFi cells. (**A**) DiFi cells were exposed to 20 nM anti-HER2 mAb 4D5 or to 20 nM each of anti-EGF receptor mAb 14E1, 108, 528, or 225 in 0.5% FBS medium for 24 h. The cells were harvested, and the chromosomal DNA was extracted and subjected to agarose gel electrophoresis. (**B**) DiFi cells were exposed to increasing concentrations of mAb 225 for 24 h, and the cells were lysed for DNA agarose gel electrophoresis analysis. (**C**) DiFi cells were exposed to 20 nM mAb 225 for the indicated times, and the cells were lysed at each time point for DNA agarose gel electrophoresis analysis. (**D**) DiFi cells were exposed to 20 nM mAb 225 for the indicated times and were cultured for additional time in mAb 225-free medium for a total of 24 h. The cells were lysed for DNA agarose gel electrophoresis analysis. Both adherent and floating cells were harvested for DNA extraction and agarose gel analysis as described in Materials and methods.

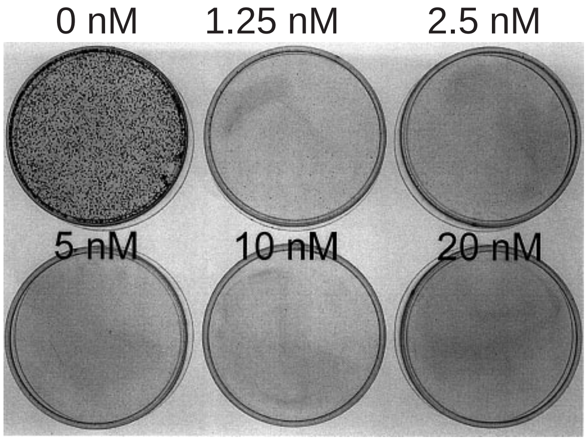

**Figure 2** Clonogenic survival of DiFi cells following treatment with anti-EGF receptor mAb 225. The cells were seeded as described in Materials and methods. On the next day, the cells were exposed to the indicated concentrations of mAb 225 and cultured in a 37°C incubator with 95% air and 5% $CO_2$ for 15 days. The cell clones were stained with crystal violet blue.

was concentration dependent (Figure 1B). The initial effective dose determined by DNA fragmentation electrophoresis analysis was greater than 5 nM of mAb 225. However, the dose required to induce apoptosis was lower (1–2 nM) with a more sensitive apoptosis ELISA analysis (data not shown). DNA fragmentation was first detectable approximately 12 h after continuous exposure of the cells to 20 nM mAb 225 (Figure 1C). However, a 1 h exposure

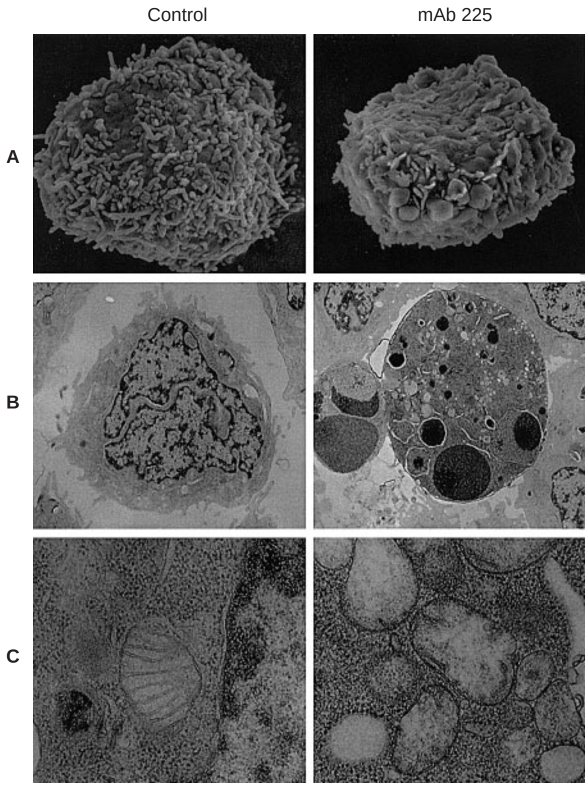

**Figure 3** Characteristic morphological changes in the cell membrane, nucleus, and mitochondria of DiFi cells following mAb 225 treatment. DiFi cells were incubated in 0.5% FBS medium containing 20 nM mAb 225 for 24 h; controls were similarly incubated without mAb 225. The cells were then harvested, fixed, and examined by scanning electron microscopy and transmission electron microscopy as described in Materials and methods. Magnified ×5000.

of the cells to 20 nM mAb 225 was sufficient to induce apoptosis that was measured 24 h later (Figure 1D). The percentage of the cells that were apoptotic at 24 h after 1 h of incubation with mAb 225 was approximately 25–30% by Hoechst (#33258) dye staining. This result was similar to the percentage of the cells that were apoptotic after being continuously exposed to mAb 225 for 24 h (data not shown).

A caveat is that the dose-dependent induction of DNA fragmentation by mAb 225 in Figure 1B does not necessarily reflect a similar pattern of end-point of cell survival. The clonogenic survival shown in Figure 2 indicates that, in a 15-d culture period, there were no differences in clone survival of the cells exposed to the serial concentrations of mAb 225 that were used in Figure 1B.

The mAb 225-induced apoptosis in DiFi cells was characterized with typical morphological changes in the cell membrane, the nuclei, and the mitochondria. Scanning electron micrography shows that untreated DiFi cells displayed features typical of colon epithelial cells, i.e. an abundance of delicate microvilli that were orderly and closely packed on the cell surface, whereas mAb 225-treated cells exhibited volume shrinkage, distortion of cell surface microvilli, and the appearance of elongated membrane blebbings (Figure 3A). Transmission electron micrography showed that untreated cells contained intact nuclei and mitochondria filled with integral cristae whereas the antibody-treated cells developed fragmented nuclei, membrane-wrapped apoptotic bodies, and swollen mitochondria (Figure 3B and 3C).

*© 2000 Cancer Research Campaign*

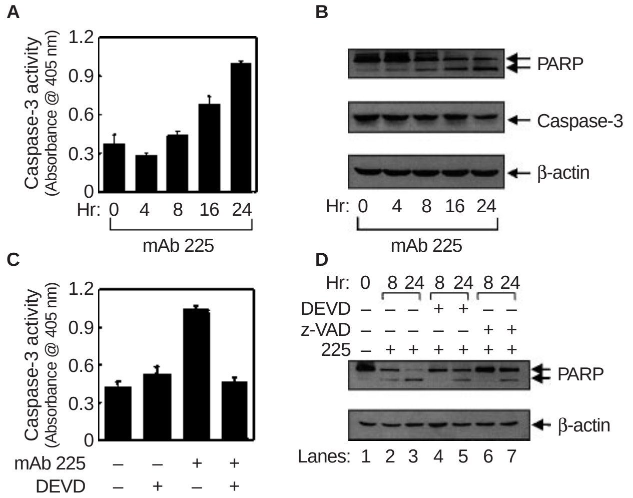

**Figure 4** Activation of caspase-3 and cleavage of PARP by mAb 225. (**A**) DiFi cells were exposed to 20 nM 225 for the indicated times. Cell lysates were prepared and subjected to an enzymatic assay for the activity of caspase-3 as described in Materials and methods. (**B**) An equal amount of protein for each sample in (**A**) was resolved by 8% SDS electrophoresis, followed by Western blot analysis with specific antibodies directed against PARP, caspase-3/CPP32, or β-actin. (**C**) DiFi cells were exposed to 20 nM mAb 225 with and without pre-incubation of the cells with 10 μM DEVD-CHO for 12 h. Cell lysates were then prepared and subjected to an enzymatic assay for caspase-3 activity. (**D**) Inhibition of mAb 225-induced PARP cleavage by caspase inhibitors. DiFi cells were pre-incubated with either 10 μM DEVD-CHO (DEVD) or 100 μM z-VAD-fmk (VAD) for 12 h. The cells were harvested 8 and 24 h after mAb 225 was added to the culture and then subjected to Western blot analysis with a specific antibody directed against PARP or β-actin.

### Role of the execution caspase-3 in mAb 225-induced apoptosis

Caspase-3 is a major execution caspase required for the characteristic DNA fragmentation (Figure 1) and morphological changes (Figure 3) associated with apoptosis (Janicke et al, 1998*a*). To demonstrate whether treatment of DiFi cells with mAb 225 triggers a cascade that results in activation of caspase-3, we measured the enzyme activity of caspase-3 following exposure of DiFi cells to mAb 225. We found a time-dependent activation of caspase-3, which was detectable between 8 h and 16 h after exposure of DiFi cells to mAb 225 (Figure 4A). This activation of caspase-3 coincided with cleavage of the 116-kDa PARP, one of well-documented substrates for activated caspase-3 (Figure 4B). The appearance of a characteristic 85-kDa PARP fragment was observed temporally with activation of caspase-3, as shown by the reduced level of the 32-kDa proenzyme due to its cleavage by its upstream caspases. Our results suggest that the apoptosis induced by mAb 225 in DiFi cells involves the caspase-3 activity.

We further explored whether there was a causal correlation of caspase-3 activation and mAb 225-induced PARP cleavage by selectively blocking caspase-3 activity with specific synthetic peptide inhibitors. When DiFi cells were exposed to the caspase-3 specific inhibitor DEVD-CHO, the caspase-3 activity was markedly inhibited 24 h after exposure of cells to mAb 225 (Figure 4C). Inhibition of caspase-3 with DEVD-CHO partially reduced the rate of PARP cleavage after mAb 225 treatment. Cleavage of PARP was detected as early as 8 h after treatment with mAb 225 alone (Figure 4D, lanes 2 and 3), but when the cells were pre-incubated with DEVD-CHO the cleavage was not detected until 24 h after treatment (Figure 4D, lanes 4 and 5). Pre-incubation of the cells with a pan-caspase inhibitor, z-VAD-fmk, more strongly prevented cleavage of PARP by mAb 225 than did DEVD-CHO (Figure 4D, lanes 6 and 7). Compared with the results of mAb 225 treatment alone, the cells pre-incubated with DEVD-CHO or z-VAD-fmk retained relatively higher levels of the 116-kDa PARP at both the 8 h and 24 h time-points (Figure 4D, lane 2 compared with lanes 4 and 6, and lane 3 compared with lanes 5 and 7, respectively), especially when the pan-caspase inhibitor z-VAD-fmk was used.

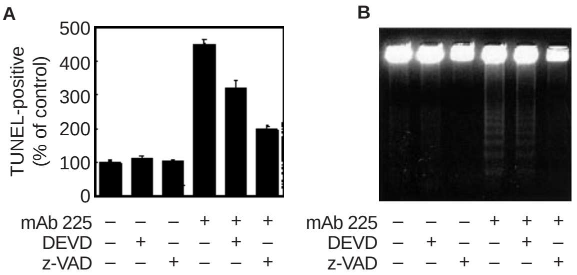

**Figure 5** Inhibition of mAb 255-induced apoptosis by caspase inhibitors. DiFi cells were pre-incubated with either 10 μM DEVD-CHO or 100 μM z-VAD-fmk for 12 h and then incubated with 20 nM mAb 225 for another 24 h. Both adherent and floating cells were collected and subjected to a TUNEL assay (**A**) and to a chromosomal DNA agarose gel electrophoresis assay (**B**).

We accordingly evaluated the effects of these caspase inhibitors on mAb 225-induced apoptosis. TUNEL assay showed that, compared with results for mAb 225 treatment alone, the relative percentage of apoptotic cells (FITC-positive cells) was lower in the cells pre-treated with DEVD-CHO or z-VAD-fmk 12 h before the cells were exposed to mAb 225 (Figure 5A), and this result was also consistent with the PARP cleavage analysis indicating that z-VAD-fmk was a stronger inhibitor than DEVD-CHO in preventing mAb 225-induced PARP cleavage and apoptosis (Figure 5B). The FITC-positive rates varied from 10–30% of total cells in several repeated experiments in our study, depending upon the intensity of FITC that was conjugated to the avidin used in the TUNEL assay. However, in each experiment, the relative fold increase of the FITC-positive rate in mAb 225-treated cells over that in untreated cells was consistent (Figure 5A). Parallel experiments analysed by chromosomal DNA agarose gel electrophoresis had similar results (Figure 5B). In these experiments, the pan-caspase inhibitor z-VAD-fmk more strongly inhibited mAb 225-induced apoptosis than did DEVD-CHO. These results indicated that activation of caspase-3 is a major step in mAb 225-induced apoptosis but also that caspase-3 may not be the only caspase contributing to the apoptosis.

### Activation of the initiation caspase-8 and caspase-9 in mAb 225-induced apoptosis

We measured the specific activities of the initiation caspases-8 and -9 following exposure of DiFi cells to mAb 225. We found a time-dependent activation of caspase-8, which was detectable between 8 h and 16 h after exposure of the cells to mAb 225 (Figure 6A). There was also an activation of caspase-9, which lagged a few hours behind the activation of caspase-8. These results indicate that both the caspase-8-initiated and the caspase-9-initiated caspase cascades are activated in the apoptosis induced by mAb

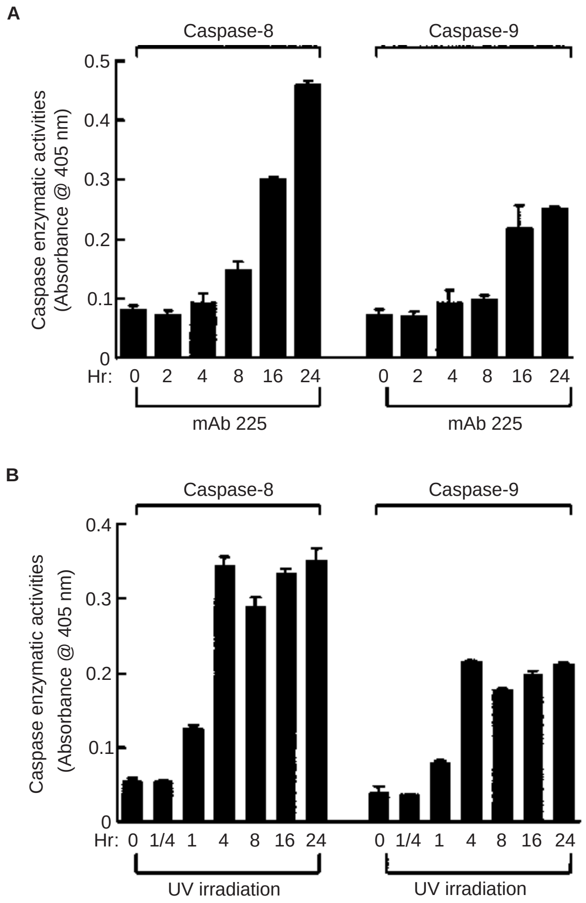

**Figure 6** Activation of caspase-8 and caspase-9 by mAb 225 and UV irradiation in DiFi cells. DiFi cells were exposed to 20 nM mAb 225 for the indicated time periods (**A**) or were UV-irradiated for 40 s, followed by regular culture for the indicated time periods (**B**). Cell lysates were prepared and subjected to enzymatic assays for the activities of caspase-8 and caspase-9 as described in Materials and methods.

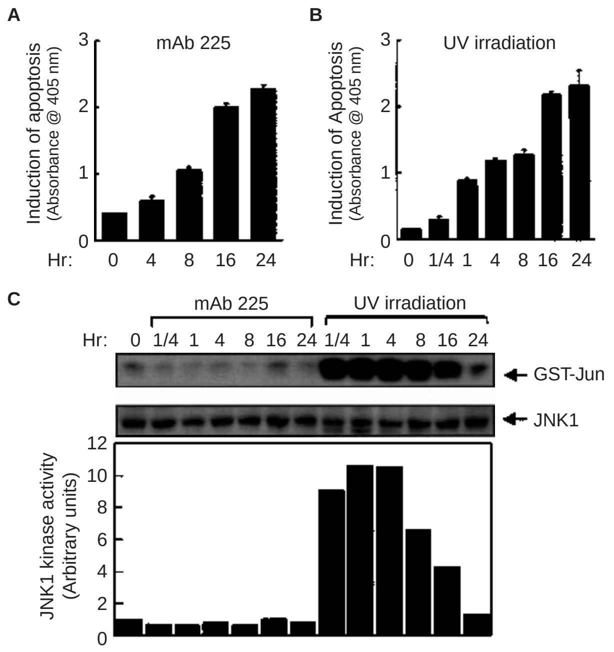

**Figure 7** Activation of JNK in UV irradiation-induced apoptosis but not in mAb 225-induced apoptosis in DiFi cells. (**A**) and (**B**) DiFi cells were exposed to 20 nM mAb 225 for the indicated time periods (**A**) or were UV-irradiated for 40 s, followed by regular culture for the indicated time periods (**B**). Cell lysate were prepared for an apoptosis ELISA as described in Materials and methods. (**C**) DiFi cells were treated as described in (**A**) and (**B**) and then harvested for JNK1 kinase activity analysis with an in vitro assay using GST-*Jun* as a substrate and for western blot analysis with antibodies directed against JNK1. The bar graph underneath was obtained by phosphoimaging analysis of GST-c-*jun* phosphorylation by JNK1.

225 in these cells. UV irradiation of DiFi cells for 40 s also activated the initiation caspases-8 and -9 (Figure 6B) and the execution caspase-3 (data not shown). However, compared with the kinetics of the caspase cascade activation by mAb 225, which showed relative delayed but steady increases in the caspase activities over 24 h, the activation of caspase-8 and caspase-9 by UV was detected 4 h shortly after the treatment and reached a plateau afterward.

### Lack of involvement of the JNK signalling pathway in mAb 225-induced apoptosis

Recent study has suggested possible interactions, under certain circumstances, between the JNKs pathway and the caspase cascade during apoptosis (Lei et al, 1998; Muhlenbeck et al, 1998; Shiah et al, 1999). JNKs participate in cellular responses to environmental stresses, apoptotic agents, and even some mitogenic stimuli (Chen et al, 1996). To investigate possible involvement of the JNK signaling pathway in mAb 225-induced apoptosis and activation of the caspase cascade in DiFi cells, we examined the JNK1 kinase activity when DiFi cells were exposed to mAb 225. Exposure of DiFi cells to either mAb 225 or UV irradiation induced apoptosis (Figure 7A and 7B). However, only the UV irradiation activated JNK1 kinase (Figure 7C). The levels of JNK1 protein remained unchanged during the exposure period to mAb 225 or UV irradiation. This result indicated that the JNK1 signaling pathway is competent in DiFi cells but that it is not involved in mAb 225-induced apoptosis in DiFi cells.

### DISCUSSION

A human-mouse chimeric version of mAb 225 (C225) is currently being evaluated in clinical trials of its antitumour activity against human cancers of the pancreas, colon, and head and neck. Ongoing studies are investigating a potential synergy between the anti-EGF receptor mAb 225-mediated disruption of EGF receptor function and conventional chemotherapy or radiation therapy (Baselga et al, 2000; Perez-Soler et al, 1998; Mendelsohn et al, 1999). Because a direct and targeted approach to cancer therapy is preferable, demonstration of the mechanism by which mAb 225 induces apoptosis specifically in DiFi colorectal cancer cells may direct us to develop new therapeutic strategies that will maximize the antitumour activity of mAb 225 by inducing programmed cell death in a broad range of human cancers without requiring combination treatment with conventional chemotherapy or radiation therapy.

In the present study, we demonstrated that apoptosis was induced by several different mAbs that recognize individual distinct epitopes on the EGF receptors on DiFi cells. This result makes it unlikely that mAb 225 induces apoptosis by cross-reacting with an

© 2000 Cancer Research Campaign unidentified ‘death’ protein rather than the EGF receptor on DiFi cell surfaces. The commitment of DiFi cells to apoptosis shortly after 1 h of exposure to mAb 225 sharply contrasts with the anti-proliferative activity of mAb 225 in many other cell lines, which normally requires over 24 h of continuous exposure of the cells to the antibody to arrest cell cycle progression, suggesting that the apoptotic activity of anti-EGF receptor mAb in DiFi cells may not be directly associated with the anti-proliferative effect of mAb 225 on many other cell lines. We also demonstrated that caspases-3, -8 and -9 are activated during mAb 225-induced apoptosis and the latter apoptosis differs from the apoptosis of DiFi cells induced by UV irradiation in that it does not involve JNK1 activity. In other studies, JNK1 activity was associated with activation of caspases by several anticancer drugs (Seimiya et al, 1997; Shiah et al, 1999). In our study, the JNK1 activity was temporally associated with the activation of caspases-3, -8 and -9 during UV irradiation-induced apoptosis, suggesting a competent JNK signalling pathway in DiFi cells during the apoptosis. However, this was not the case in the activation of the caspase cascade during mAb 225-induced apoptosis in these cells.

An understanding of caspase regulation is intimately linked to the ability of researchers to rationally manipulate apoptosis for therapeutic application to human cancer. Caspases are constitutively expressed as latent proenzymes in living cells. Two initiation pathways of caspase cascade during apoptosis have been described (Thornberry and Lazebnik, 1998; Ashkenazi and Dixit, 1998; Green and Reed, 1998; Sun et al, 1999). The first pathway is the caspase-8 initiated apoptosis induced by death receptors, such as Fas or tumour necrosis factor (TNF) receptors (Boldin et al, 1996; Muzio et al, 1996; 1998; Yang et al, 1998); the second pathway involves the complex of cytochrome *c*, Apaf-1 and caspase-9 following stimulation of the cells with diverse proapoptotic signals that converge at the mitochondrial level and provoke the translocation of cytochrome *c* from the mitochondria to the cytoplasm (Kluck et al, 1997; Adachi et al, 1997; Kharbanda et al, 1997; Kim et al, 1997; Bossy-Wetzel et al, 1998). The characteristic induction of apoptosis by anti-EGF receptor mAb is reminiscent of the apoptosis induced by anti-*fas*/CD95 mAb (Itoh et al, 1991). We examined the possible involvement of the *fas*-mediated apoptotic pathway in mAb 225-induced apoptosis. Exposure of DiFi cells to an anti-*fas* mAb did not trigger apoptosis, suggesting that DiFi cells do not constitutively express functional *fas* (data not shown). The apoptosis induced by the anti-EGF receptor mAb has so far been observed only in DiFi colon cancer cells. In most tumour cell lines studied, blockade of the EGF receptor with mAb has resulted in cell cycle arrest rather than cell death. Two possible mechanisms by which anti-EGF receptor mAbs induce apoptosis are being considered. The first possibility is that the DiFi cells may be genetically defective in certain components that normally inhibit induction of apoptosis upon EGF receptor blockade. Alternatively, the EGF receptor in DiFi cells may link to molecule(s) that have apoptosis-triggering activity, and binding of the receptor by mAb may result in a release or activation of such molecule(s). Both caspase-8 and caspase-9 may initiate caspase cascade and thereby induce apoptosis, however, we speculate that the observed activation of caspase-9 in DiFi cells after mAb 225 treatment could be a consequence of mAb 225-induced activation of caspase-8 through a mechanism similar to *fas*-mediated activation of caspase-8 and caspase-9. Caspase-8 activated by *fas* pathway can cleave Bid (a BH3 domain-containing proapoptotic Bcl-2 family member), and the truncated Bid will then translocate to the mitochondria and induce the release of cytochrome *c* and subsequent activation of caspase-9 (Li et al, 1998). Our data showing that the kinetics of caspase-9 activation lagged a few hours behind the activation of caspase-8 support this speculation (Figure 6).

Among the known execution caspases (caspase-3, -6 and -7, etc), caspase-3 is the major caspase. Induction of apoptosis in caspase-3-defective MCF-7 human breast cancer cells was accompanied by cleavage of PARP, Rb, and DNA-PK without concurrent appearance of the characteristic DNA internucleosomal degradation and the characteristic morphological changes associated with apoptosis; however, introduction of caspase-3 cDNA into the MCF-7 cells restored α-fodrin cleavage and membrane blebbing (Janicke et al, 1998*a*; 1998*b*). The characteristic induction of DNA laddering and membrane blebbing in DiFi cells that we observed following treatment with mAb 225 (Figures 1 and 3) led us to examine the involvement of caspase-3 in mAb 225-induced apoptosis in DiFi cells. Our data indicate that caspase-3 is a major caspase contributing to apoptosis in DiFi cells. In addition, this pathway also involves activation of caspase-8 and caspase-9. Inhibition of caspase-3 and pan-caspase activities by specific inhibitors strongly reduced induction of the apoptosis with concomitant inhibition of PARP cleavage and DNA fragmentation.

Caspases contribute to apoptosis in a tissue- or cell type-specific or death signal-specific manner. Caspase-3-disrupted cells that were highly resistant to apoptosis induced by UV irradiation or osmotic shock were still sensitive to apoptosis induced by γ-irradiation or heat shocks (Woo et al, 1998). The demonstration of the involvement of the caspase cascade (the initiation caspases-8 and -9 and the execution caspase-3) in mAb 225-induced apoptosis provides important insights for us to further pursue the mechanism.

In summary, we demonstrated that apoptosis could be induced in DiFi colon cancer cells by a variety of anti-EGF receptor mAbs that bind to different epitopes on the receptor. This apoptosis is characterized by classical DNA internucleosomal degradation and typical caspase-mediated morphological changes, and this apoptosis involves activation of caspase-8- and caspase-9-initiated caspase cascade. We are currently investigating the biochemical pathway(s) by which mAb 225 induces activation of the caspase cascade, thereby triggering apoptosis.

### ACKNOWLEDGEMENTS

This work was supported by the NIH grant CA42060, the NCI Cancer Center core grant CA16672 and an MD Anderson Cancer Center Start-up fund (to ZF). The authors are grateful to Dr C Bucana and Mr K Dunner Jr (high-resolution electron microscopy core facility) and Mrs K Ramirez (flow cytometry core facility) for technical assistance with the manuscript, and to Mr M Worley (the Department of Scientific Publications) for editorial assistance with the manuscript. M Schmidt was supported by a fellowship from the Ernst Schering Research Foundation, Berlin, Germany.

### REFERENCES

Adachi S, Cross AR, Babior BM and Gottlieb RA (1997) Bcl-2 and the outer mitochondrial membrane in the inactivation of cytochrome c during Fas-mediated apoptosis. *J Biol Chem* **272**: 21878–21882

© 2000 Cancer Research Campaign 

Ashkenazi A and Dixit VM (1998) Death receptors: signaling and modulation. *Science* **281**: 1305–1308
Baselga J, Mendelsohn J, Kim YM and Pandiella A (1996) Autocrine regulation of membrane transforming growth factor-alpha cleavage. *J Biol Chem* **271**: 3279–3284
Baselga J, Pfister D, Cooper MR, Cohen R, Burtness B, Bos M, D'Andrea G, Seidman A, Norton L, Gunnett K, Falcey J, Anderson V, Waksal H and Mendelsohn J (2000) Phase I studies of anti-epidermal growth factor receptor chimeric antibody C225 alone and in combination with cisplatin. *J Clin Oncol*: *In press*
Boldin MP, Goncharov TM, Goltsev YV and Wallach D (1996) Involvement of MACH, a novel MORT1/FADD-interacting protease, in Fas/APO-1- and TNF receptor-induced cell death. *Cell* **85**: 803–815
Bossy-Wetzel E, Newmeyer DD and Green DR (1998) Mitochondrial cytochrome c release in apoptosis occurs upstream of DEVD-specific caspase activation and independently of mitochondrial transmembrane depolarization. *EMBO J* **17**: 37–49
Bump NJ, Hackett M, Hugunin M, Seshagiri S, Brady K, Chen P, Ferenz C, Franklin S, Ghayur T and Li P (1995) Inhibition of ICE family proteases by baculovirus antiapoptotic protein p35. *Science* **269**: 1885–1888
Chen YR, Wang X, Templeton D, Davis RJ and Tan TH (1996) The role of c-Jun N-terminal kinase (JNK) in apoptosis induced by ultraviolet C and gamma radiation. Duration of JNK activation may determine cell death and proliferation. *J Biol Chem* **271**: 31929–31936
Chou JL, Fan Z, DeBlasio T, Koff A, Rosen N and Mendelsohn J (1999) Constitutive overexpression of cyclin D1 in human breast epithelial cells does not prevent G1 arrest induced by deprivation of epidermal growth factor. *Breast Cancer Res Treat* **55**: 267–283
Cohen GM (1997) Caspases: the executioners of apoptosis. *Biochem J* **326 (Pt 1)**: 1–16
Cowley GP, Smith JA and Gusterson BA (1986) Increased EGF receptors on human squamous carcinoma cell lines. *Br J Cancer* **53**: 223–229
Ellis HM and Horvitz HR (1986) Genetic control of programmed cell death in the nematode *C. elegans. Cell* **44**: 817–829
Ennis BW, Valverius EM, Bates SE, Lippman ME, Bellot F, Kris R, Schlessinger J, Masui H, Goldenberg A, Mendelsohn J and Dickson RB (1989) Anti-epidermal growth factor receptor antibodies inhibit the autocrine-stimulated growth of MDA-468 human breast cancer cells. *Mol Endocrinol* **3**: 1830–1838
Fan Z and Mendelsohn J (1998) Therapeutic application of anti-growth factor receptor antibodies. *Curr Opin Oncol* **10**: 67–73
Fan Z, Baselga J, Masui H and Mendelsohn J (1993*a*) Antitumour effect of anti-epidermal growth factor receptor monoclonal antibodies plus cis-diamminedichloroplatinum on well established A431 cell xenografts. *Cancer Res* **53**: 4637–4642
Fan Z, Masui H, Altas I and Mendelsohn J (1993b) Blockade of epidermal growth factor receptor function by bivalent and monovalent fragments of 225 anti-epidermal growth factor receptor monoclonal antibodies. *Cancer Res* **53**: 4322–4328
Fan Z, Mendelsohn J, Masui H and Kumar R (1993c) Regulation of epidermal growth factor receptor in NIH3T3/HER14 cells by antireceptor monoclonal antibodies. *J Biol Chem* **268**: 21073–21079
Fan Z, Lu Y, Wu X and Mendelsohn J (1994) Antibody-induced epidermal growth factor receptor dimerization mediates inhibition of autocrine proliferation of A431 squamous carcinoma cells. *J Biol Chem* **269**: 27595–27602
Fan Z, Shang BY, Lu Y, Chou JL and Mendelsohn J (1997) Reciprocal changes in $p27^{Kip1}$ and $p21^{Cip1}$ in growth inhibition mediated by blockade or overstimulation of epidermal growth factor receptors. *Clin Cancer Res* **3**: 1943–1948
Gill GN, Kawamoto T, Cochet C, Le A, Sato JD, Masui H, McLeod C and Mendelsohn J (1984) Monoclonal anti-epidermal growth factor receptor antibodies which are inhibitors of epidermal growth factor binding and antagonists of epidermal growth factor binding and antagonists of epidermal growth factor-stimulated tyrosine protein kinase activity. *J Biol Chem* **259**: 7755–7760
Green DR (1998) Apoptotic pathways: the roads to ruin. *Cell* **94**: 695–698
Green DR and Reed JC (1998) Mitochondria and apoptosis. *Science* **281**: 1309–1312
Gross ME, Zorbas MA, Danels YJ, Garcia R, Gallick GE, Olive M, Brattain MG, Boman BM and Yeoman LC (1991) Cellular growth response to epidermal growth factor in colon carcinoma cells with an amplified epidermal growth factor receptor derived from a familial adenomatous polyposis patient. *Cancer Res* **51**: 1452–1459
Hendler FJ and Ozanne BW (1984) Human squamous cell lung cancers express increased epidermal growth factor receptors. *J Clin Invest* **74**: 647–651
Hofer DR, Sherwood ER, Bromberg WD, Mendelsohn J, Lee C and Kozlowski JM (1991) Autonomous growth of androgen-independent human prostatic carcinoma cells: role of transforming growth factor alpha. *Cancer Res* **51**: 2780–2785
Ishitoya J, Toriyama M, Oguchi N, Kitamura K, Ohshima M, Asano K and Yamamoto T (1989) Gene amplification and overexpression of EGF receptor in squamous cell carcinomas of the head and neck. *Br J Cancer* **59**: 559–562
Itoh N, Yonehara S, Ishii A, Yonehara M, Mizushima S, Sameshima M, Hase A, Seto Y and Nagata S (1991) The polypeptide encoded by the cDNA for human cell surface antigen Fas can mediate apoptosis. *Cell* **66**: 233–243
Janicke RU, Sprengart ML, Wati MR and Porter AG (1998*a*) Caspase-3 is required for DNA fragmentation and morphological changes associated with apoptosis. *J Biol Chem* **273**: 9357–9360
Janicke RU, Ng P, Sprengart ML and Porter AG (1998*b*) Caspase-3 is required for alpha-fodrin cleavage but dispensable for cleavage of other death substrates in apoptosis. *J Biol Chem* **273**: 15540–15545
Karnes WJ, Walsh JH, Wu SV, Kim RS, Martin MG, Wong HC, Mendelsohn J, Park JG and Cuttitta F (1992) Autonomous proliferation of colon cancer cells that coexpress transforming growth factor alpha and its receptor. Variable effects of receptor-blocking antibody. *Gastroenterology* **102**: 474–485
Kharbanda S, Pandey P, Schofield L, Israels S, Roncinske R, Yoshida K, Bharti A, Yuan ZM, Saxena S, Weichselbaum R, Nalin C and Kufe D (1997) Role for Bcl-xL as an inhibitor of cytosolic cytochrome C accumulation in DNA damage-induced apoptosis. *Proc Natl Acad Sci U S A* **94**: 6939–6942
Kim CN, Wang X, Huang Y, Ibrado AM, Liu L, Fang G and Bhalla K (1997) Overexpression of Bcl-X(L) inhibits Ara-C-induced mitochondrial loss of cytochrome c and other perturbations that activate the molecular cascade of apoptosis. *Cancer Res* **57**: 3115–3120
Kluck RM, Bossy-Wetzel E, Green DR and Newmeyer DD (1997) The release of cytochrome c from mitochondria: a primary site for Bcl-2 regulation of apoptosis. *Science* **275**: 1132–1136
Lee M, Draoui M, Zia F, Gazdar A, Oie H, Bepler G, Bellot F, Tarr C, Kris R and Moody TW (1992) Epidermal growth factor receptor monoclonal antibodies inhibit the growth of lung cancer cell lines. *J Natl Cancer Inst Monogr* **13**: 117–123
Lei W, Yu R, Mandlekar S and Kong AN (1998) Induction of apoptosis and activation of interleukin 1beta-converting enzyme/Ced-3 protease (caspase-3) and c-Jun NH2-terminal kinase 1 by benzo(a)pyrene. *Cancer Res* **58**: 2102–2106
Li H, Zhu H, Xu CJ and Yuan J (1998) Cleavage of BID by caspase 8 mediates the mitochondrial damage in the Fas pathway of apoptosis. *Cell* **94**: 491–501
Masui H, Boman BM, Hyman J, Castro L and Mendelsohn J (1991) Treatment with anti-EGF receptor monoclonal antibody causes regression of DiFi human colorectal carcinoma xenografts. *Proc Am Assoc Cancer Res* **32**: 394
Mendelsohn J, Shin DM, Donato N, Khuri F, Radinsky R, Glisson BS, Shin HJ, Metz E, Pfister D, Perez-Soler R, Lawhorn K, Matsumoto T, Gunnett K, Falcey J, Waksal H and Hong WK (1999) A phase I study of chimerized anti-epidermal growth factor receptor (EGFR) monoclonal antibody, C225, in combination with cisplatin (CDDP) in patients (pts) with recurrent head and neck squamous cell carcinoma (SCC). *Proc Am Soc Clin Oncol* **18**: 389a (abstract 1502)
Modjtahedi H, Eccles S, Box G, Styles J and Dean C (1993) Immunotherapy of human tumour xenografts overexpressing the EGF receptor with rat antibodies that block growth factor-receptor interaction. *Br J Cancer* **67**: 254–261
Muhlenbeck F, Haas E, Schwenzer R, Schubert G, Grell M, Smith C, Scheurich P and Wajant H (1998) TRAIL/Apo2L activates c-Jun NH2-terminal kinase (JNK) via caspase-dependent and caspase-independent pathways. *J Biol Chem* **273**: 33091–33098
Muzio M, Chinnaiyan AM, Kischkel FC, O'Rourke K, Shevchenko A, Ni J, Scaffidi C, Bretz JD, Zhang M, Gentz R, Mann M, Krammer PH, Peter ME and Dixit VM (1996) FLICE, a novel FADD-homologous ICE/CED-3-like protease, is recruited to the CD95 (Fas/APO-1) death-inducing signaling complex. *Cell* **85**: 817–827
Muzio M, Stockwell BR, Stennicke HR, Salvesen GS and Dixit VM (1998) An induced proximity model for caspase-8 activation. *J Biol Chem* **273**: 2926–2930
Neal DE, Marsh C, Bennett MK, Abel PD, Hall RR, Sainsbury JR and Harris AL (1985) Epidermal-growth-factor receptors in human bladder cancer: comparison of invasive and superficial tumours. *Lancet* **1**: 366–368
Ozanne B, Richards CS, Hendler F, Burns D and Gusterson B (1986) Over-expression of the EGF receptor is a hallmark of squamous cell carcinomas. *J Pathol* **149**: 9–14
Peng D, Fan Z, Lu Y, DeBlasio T, Scher H and Mendelsohn J (1996) Anti-epidermal growth factor receptor monoclonal antibody 225 up-regulates $p27^{KIP1}$ and induces G1 arrest in prostatic cancer cell line DU145. *Cancer Res* **56**: 3666–3669

 © 2000 Cancer Research Campaign

Perez-Soler R, Shin D, Donato N, Radinsky R, Khuri F, Glisson BS, Shin H, Matsumoto T, Lawhorn K, Hong WK and Mendelsohn J (1998) Tumour studies in patients with head and neck cancer treated with humanized anti-epidermal growth factor receptor (EGFR) monoclonal antibody C225 in combination with cisplatin. *Proc Am Soc Clin Oncol* **17**: 393a (abstract 1514)

Perrotte P, Matsumoto T, Inoue K, Kuniyasu H, Eve BY, Hicklin DJ, Radinsky R and Dinney CP (1999) Anti-epidermal growth factor receptor antibody C225 inhibits angiogenesis in human transitional cell carcinoma growing orthotopically in nude mice. *Clin Cancer Res* **5**: 257–265

Prewett M, Rothman M, Waksal H, Feldman M, Bander NH and Hicklin DJ (1998) Mouse-human chimeric anti-epidermal growth factor receptor antibody C225 inhibits the growth of human renal cell carcinoma xenografts in nude mice. *Clin Cancer Res* **4**: 2957–2966

Sainsbury JR, Malcolm AJ, Appleton DR, Farndon JR and Harris AL (1985) Presence of epidermal growth factor receptor as an indicator of poor prognosis in patients with breast cancer. *J Clin Pathol* **38**: 1225–1228

Sato JD, Kawamoto T, Le AD, Mendelsohn J, Polikoff J and Sato GH (1983) Biological effects in vitro of monoclonal antibodies to human epidermal growth factor receptors. *Mol Biol Med* **1**: 511–529

Schmidt M, Vakalopoulou E, Schneider DW and Wels W (1997) Construction and functional characterization of scFv(14E1)-ETA – a novel, highly potent antibody-toxin specific for the EGF receptor. *Br J Cancer* **75**: 1575–1584

Seimiya H, Mashima T, Toho M and Tsuruo T (1997) c-Jun NH2-terminal kinase-mediated activation of interleukin-1β converting enzyme/CED-3-like protease during anticancer drug-induced apoptosis. *J Biol Chem* **272**: 4631–4636

Shiah SG, Chuang SE, Chau YP, Shen SC and Kuo ML (1999) Activation of c-Jun NH2-terminal kinase and subsequent CPP32/Yama during topoisomerase inhibitor β-lapachone-induced apoptosis through an oxidation-dependent pathway. *Cancer Res* **59**: 391–398

Stampfer MR, Pan CH, Hosoda J, Bartholomew J, Mendelsohn J and Yaswen P (1993) Blockage of EGF receptor signal transduction causes reversible arrest of normal and immortal human mammary epithelial cells with synchronous reentry into the cell cycle. *Exp Cell Res* **208**: 175–188

Sun XM, MacFarlane M, Zhuang J, Wolf BB, Green DR and Cohen GM (1999) Distinct caspase cascades are initiated in receptor-mediated and chemical-induced apoptosis. *J Biol Chem* **274**: 5053–5060

Susin SA, Lorenzo HK, Zamzami N, Marzo I, Snow BE, Brothers GM, Mangion J, Jacotot E, Costantini P, Loeffler M, Larochette N, Goodlett DR, Aebersold R, Siderovski DP, Penninger JM and Kroemer G (1999) Molecular characterization of mitochondrial apoptosis-inducing factor. *Nature* **397**: 441–446

Tewari M, Quan LT, O'Rourke K, Desnoyers S, Zeng Z, Beidler DR, Poirier GG, Salvesen GS and Dixit VM (1995) Yama/CPP32 beta, a mammalian homolog of CED-3, is a CrmA-inhibitable protease that cleaves the death substrate poly(ADP-ribose) polymerase. *Cell* **81**: 801–809

Thornberry NA and Lazebnik Y (1998) Caspases: enemies within. *Science* **281**: 1312–1316

Ullrich A and Schlessinger J (1990) Signal transduction by receptors with tyrosine kinase activity. *Cell* **61**: 203–212

Untawale S, Zorbas MA, Hodgson CP, Coffey RJ, Gallick GE, North SM, Wildrick DM, Olive M, Blick M, Yeoman LC and Boman BM (1993) Transforming growth factor-alpha production and autoinduction in a colorectal carcinoma cell line (DiFi) with an amplified epidermal growth factor receptor gene. *Cancer Res* **53**: 1630–1636

Van de Vijver MJ, Kumar R and Mendelsohn J (1991) Ligand-induced activation of A431 cell epidermal growth factor receptors occurs primarily by an autocrine pathway that acts upon receptors on the surface rather than intracellularly. *J Biol Chem* **266**: 7503–7508

Veale D, Ashcroft T, Marsh C, Gibson GJ and Harris AL (1987) Epidermal growth factor receptors in non-small cell lung cancer. *Br J Cancer* **55**: 513–516

Wersall P, Ohlsson I, Biberfeld P, Collins VP, von Krusenstjerna S, Larsson S, Mellstedt H and Boethius J (1997) Intratumoural infusion of the monoclonal antibody, mAb 425, against the epidermal-growth-factor receptor in patients with advanced malignant glioma. *Cancer Immunol Immunother* **44**: 157–164

Woo M, Hakem R, Soengas MS, Duncan GS, Shahinian A, Kagi D, Hakem A, McCurrach M, Khoo W, Kaufman SA, Senaldi G, Howard T, Lowe SW and Mak TW (1998) Essential contribution of caspase 3/CPP32 to apoptosis and its associated nuclear changes. *Genes Dev* **12**: 806–819

Wu X, Fan Z, Masui H, Rosen N and Mendelsohn J (1995) Apoptosis induced by an anti-epidermal growth factor receptor monoclonal antibody in a human colorectal carcinoma cell line and its delay by insulin. *J Clin Invest* **95**: 1897–1905

Wu X, Rubin M, Fan Z, DeBlasio T, Soos T, Koff A and Mendelsohn J (1996) Involvement of $p27^{KIP1}$ in G1 arrest mediated by an anti-epidermal growth factor receptor monoclonal antibody. *Oncogene* **12**: 1397–1403

Xiang J, Chao DT and Korsmeyer SJ (1996) BAX-induced cell death may not require interleukin 1β-converting enzyme-like proteases. *Proc Natl Acad Sci U S A* **93**: 14559–14563

Yang X, Chang HY and Baltimore D (1998) Autoproteolytic activation of pro-caspases by oligomerization. *Mol Cell* **1**: 319–325

Ye D, Mendelsohn J and Fan Z (1999) Augmentation of a humanized anti-HER2 mAb 4D5 induced growth inhibition by a human-mouse chimeric anti-EGF receptor mAb C225. *Oncogene* **18**: 731–738

© 2000 Cancer Research Campaign
